# Experiences and Perceptions of Crisis Text Services: Interview Study Among Young Adults With Suicidal Ideation

**DOI:** 10.2196/89836

**Published:** 2026-08-26

**Authors:** Kate LaForge, Alan R Teo, Lauren Denneson, Jennifer A Hoffmann, Peter C Britton, Ashley A Foster

**Affiliations:** 1 Center to Improve Veteran Involvement in Care (CIVIC), VA Portland Health Care System, 3710 SW US Veterans Hospital Rd, Portland, OR, 97239, United States, 1 6208209939; 2 Department of Psychiatry, Oregon Health & Science University, Portland, OR, United States; 3 Division of Emergency Medicine, Ann & Robert H. Lurie Children's Hospital of Chicago, Chicago, IL, United States; 4 Center of Excellence (CoE) for Suicide Prevention, VA Finger Lakes Healthcare System, Canandaigua, NY, United States; 5 Department of Emergency Medicine, University of California, San Francisco, 550 16th Street, PO Box 0649, San Francisco, CA, 94143, United States

**Keywords:** mental health, mental health services, suicidal ideation, text messaging, young adult, health care technology

## Abstract

**Background:**

Suicide remains a leading cause of death among young adults aged 18 to 25 years. Young adults experiencing suicidal ideation (SI) are increasingly using crisis text services (CTSs), a free and accessible option for crisis intervention. Little is known about CTSs from the young adult perspective.

**Objective:**

This study aimed to characterize young adults’ experiences with and perceptions of CTSs for SI.

**Methods:**

We conducted in-depth interviews, by phone, Zoom, or text, with young adults (n=39) in the United States who had a lifetime history of SI. Participants included those who had or had not engaged with CTSs for SI. Semistructured interviews were conducted from January to July 2024. The data were analyzed using a modified grounded theory approach.

**Results:**

We constructed 5 key themes to characterize young adults’ perceptions of and experiences with CTSs for SI. Young adults perceived CTSs as a unique component of their mental health crisis management. They appreciated CTSs’ technological features, particularly the privacy they provided and the ability to reflect on and edit responses. However, they expressed dissatisfaction with the nonspecific nature of many CTS interactions. The perceived anonymity of CTSs served multiple functions, both as a motivator for CTS use and as a potential point of vulnerability, should it be lost during a CTS interaction. Participants’ perceptions of CTSs’ impact varied; some viewed them as beneficial, whereas others reported neutral or inconsistent effects over time.

**Conclusions:**

Among young adults with SI, CTSs are a key yet imperfect resource. Quality improvement and evaluation efforts may be needed to understand how responders can better tailor responses to improve conversational quality and consistency for young adult texters.

## Introduction

Suicide continues to be a leading cause of death among young adults [1]. In 2023, 1 in 5 (20.4%) US high school students seriously considered attempting suicide, and nearly 1 in 10 (9.5%) attempted suicide [2]. Rates are even higher among lesbian, gay, bisexual, transgender/transsexual, queer, and other minority sexual orientations and gender identities (LGBTQ+) students—41% considered attempting suicide (compared to 13% of heterosexual students) and 20% attempted suicide (compared to 6% of heterosexual students) [2]. Suicide risk also varies by race, and rates of suicide among Black and Asian or Pacific Islander young adults have recently increased, while rates of suicide death among American Indian or Alaska Native young adults remain the highest among any racial group [3].

Suicidal behavior is often preceded by suicidal ideation (SI), and providing mental health support to young adults with SI may help deter suicide. Young adults use a variety of support sources when experiencing SI, including friends or family, mental health professionals, school counselors, urgent care, emergency departments, psychiatric-specific emergency facilities, or mental health hotlines [4-7], which together comprise the young adult’s care ecosystem. Many young adults with SI do not seek professional help [8], often due to stigma, beliefs in self-reliance, or ambivalence toward treatment needs [4,9,10].

Health systems often struggle to meet the needs of young adults; pediatric and young adult psychiatric appointments continue to be challenging to schedule [11], with median wait times of more than 43 days [12]. Due to these challenges and difficulties with adequate insurance coverage for mental health care [13], EDs often serve as the health care safety net for suicide-related symptoms, with a 5-fold increase in these visits by adolescents and young adults from 2011 to 2020, where young adults may experience prolonged wait times, have limited access to resources, and worsening mental health symptoms [5,14].

Given these barriers to accessing in-person mental health services, alternatives are needed, and crisis text services (CTSs) represent a key potential source of support. Available 24/7 through text messaging, a CTS encounter involves initiation from a texter and a response from a CTS counselor. Few CTS conversations lead to escalation; for Crisis Text Line, fewer than 1% of conversations result in a response from emergency medical services (EMS) or law enforcement [15].

In the United States, the recently established federal crisis line, 988 [16], and Crisis Text Line are among the largest CTSs. Volume for both is high; between July 2022 and December 2024, 988 received 2.9 million texts, and Crisis Text Line reported hosting 1.3 million text conversations in 2022, 24% of which involved young adults aged 18 to 24 years [17]. Given that low socioeconomic status often complicates suicide-related help-seeking broadly [18], CTSs are frequently used by rural and low-income populations, as they offer a cost-free, remote option for care [19]. Increasing evidence indicates that CTSs may decrease short-term suicide-related distress [20-24]. Others have reported a mixed impact of CTSs on well-being, including positive and negative experiences, and issues with formulaic speech from counselors [24-27].

Survey and computational approaches have been used to understand the crisis text user perspective, finding that users may view CTSs as a safe space for communicating about SI or as an alternative to other professional services [28]. CTSs offer a sense of anonymity [29], control [24,28], and flexibility [30]. Users of crisis text or chat services are more likely to consider a conversation successful when they feel that CTS counselors care for them [21,22], are attuned to the conversational trajectory, and write longer messages [31].

Qualitative understanding of CTSs is limited. One UK-based study found that when using CTSs, young adults appreciated 24/7 service availability, solution-focused support, and a person-centric approach [26]. A New Zealand-based study found that young adults appreciated the control and familiarity of texting and the ability to text without their parents knowing [30]. However, young adults’ experiences and perceptions of CTSs in the United States remain less well understood, including how they situate CTSs within their mental health ecosystems; the technological and conversational features that encourage or discourage their use; how they evaluate the quality and usefulness of CTSs; and their perceived impact.

To fill these gaps, we conducted a qualitative study with 39 young adults with SI in Oregon. We aimed to answer the following research question: *What are young adults’ perceptions of and experiences with considering or engaging with CTS when experiencing SI?* Study findings offer insight into how young adults situate and engage with CTSs, the factors that motivate or discourage CTS use, and the ways in which they are affected by engagement, crucially illuminating diverse experiences among young adults with SI in the United States.

## Methods

### Research Design

The study data came from a mixed methods study exploring how young adults use crisis services for SI. The broader study included a survey and semistructured interviews. This article draws from interview data only.

### Ethical Considerations

This study was reviewed and approved by the University of California San Francisco Institutional Review Board (22‐37903). Informed consent was obtained from participants prior to each interview. Participants received US $30 for study participation. Participant privacy was maintained by collecting only necessary participant information and securing data files in a password-protected location.

### Participants

Eligibility included residing in Oregon, United States, being aged 18 to 25 years, and having had suicidal thoughts.

We recruited participants from August 2023 to July 2024 via Meta and Reddit, community partners, and the survey portion of the broader study. The study lead (KL) had no contact with participants prior to study recruitment. Participants completed an eligibility survey hosted on Qualtrics, which included demographic and CTS use items. A total of 161 individuals completed the eligibility screening. Of 90 eligible individuals approached to participate, 49% (n=44) agreed, 5 dropped out, resulting in a final sample of 39 participants. Demographic items were collected to diversify and understand the study sample. Participants were selected and contacted based on diversifying the sample in terms of gender, sex assigned at birth, race, and geography.

Given the sensitive nature of the study’s content, safety protocols were developed with input from team members with psychiatric specialization (ART) to be used if a participant revealed active SI during the interview. Within-interview protocols varied by interview mode: in the event of severe SI, the interviewer would stop the interview and provide a warm hand-off to 988 (via 3-way call for phone or text, or by remaining on Zoom while the participant called). Procedures were described in the consent form and reviewed before each interview. No participant disclosed SI requiring the activation of these steps.

### Data Collection

We conducted 1 pilot interview in each interview mode (phone, Zoom, text message) before data collection. We developed a semistructured interview guide by drawing from literature and team expertise with crisis lines. Interview domains included experiences with SI, help-seeking, crisis services, and crisis texting. Prompts and probes were used to gain depth and clarify responses. Consistent with grounded theory analysis [32], question categories and items were added to the interview guide as interviews progressed, including privacy and control, emerging adulthood, and intersectionality (see Multimedia Appendix 1 for the final interview guide).

From January to July 2024, the lead author (KL) conducted semistructured one-on-one interviews with participants (n=39). During the interviews, KL identified herself as a doctoral student and a volunteer crisis text counselor. KL’s experience as a crisis text counselor informed many aspects of the study. The interview guide domain areas were shaped by her experience as a volunteer counselor, as well as the construction of interview guide items related to the technological interface. Prompts and probes used throughout the interview guide were likely influenced by her level of familiarity with the CTS service but were used to elicit specific examples (eg, “Could you describe an example of when crisis texting felt convenient?”), based on the grounded theory interviewing tradition. To mitigate bias, KL wrote analytical memos after each interview, debriefed with monthly study advisors, and presented ongoing findings and sought feedback from multiple peer research groups (none of whom were crisis text counselors).

The interviews lasted between 25 and 136 minutes (average 78, SD 28.83 min) and were conducted in English via phone (n=12), Zoom (n=8), or text message (n=19). Participants’ chosen interview modes are listed in Multimedia Appendix 2. The text message interview option was provided to increase study accessibility and has been recommended for research on sensitive topics [33]. All interviews followed a similar format: they were scheduled at an agreed-upon time, they were conducted synchronously, and when interviews went longer than 90 minutes, KL offered to continue the interview at a future time (1 participant accepted this option).

We stopped collecting data based on (1) a priori study plans informed by our extensive experience conducting in-depth interviews; (2) approaching exhaustive saturation of emergent properties within categories [32]; and (3) the research team’s determination that sufficient data had been collected to support rich, analytic, and qualitative insights. Informed consent was obtained verbally prior to each interview. Participants received US $30 for participation, which was sent digitally.

### Data Analysis

Data analysis was undertaken by a sociologist with training and experience in qualitative methods, and volunteer work as a crisis text counselor (KL) and an emergency medicine physician (AAF) with qualitative experience and was overseen by a psychiatrist with qualitative experience (ART). Analytical refinements were conducted with other physician-scientists (JH), a clinical psychologist (PCB), and a social psychologist (LD) to provide a multidisciplinary perspective. Phone and Zoom interviews were transcribed verbatim, and text message interviews were used in their original form. Participants were assigned pseudonyms, which are reported in this article. Quotations from text message interviews are unedited and presented verbatim throughout this article.

We analyzed data using a modified grounded theory approach [32]. Grounded theory steps were followed throughout data collection (eg, using a semistructured interview guide and revising it throughout data collection) and preliminary analysis (eg, using open coding, focused coding, the development of categories, analytical memo writing, and the use of the constant comparative method). This process was modified primarily in the later analytical stages by using conceptual categories to develop thematic understanding (rather than building abstract theory). This decision was made to produce actionable findings for CTS provider audiences.

An audit trail was written and updated throughout the analytical process [34]. Data analysis began with transcribing and reading transcripts. Data were analyzed using 2 rounds of coding: open coding and focused coding. Open coding involved identifying and labeling data deemed interesting, novel, or potentially analytically significant. Open codes were developed inductively, and focused codes were developed by promoting and combining open codes deemed salient across the dataset, relevant to suicide-related help-seeking and crisis texting, and deemed to hold potential for analytical development. All data were initially coded by the lead author (KL). Code applications were reviewed by a second analyst (AAF), and code applications were refined in ongoing weekly meetings. Constant comparison was used throughout the entire analytical process. For example, analytical memos, incidents (eg, law enforcement involvement after an SI disclosure), and code applications (eg, applications of “conceptualizing privacy”) were compared across transcripts. Analytical memos and codes were refined through this constant comparison process. Negative case analysis was also used by actively seeking quotes that contradicted one another.

We formed categories by analytically grouping focused codes that fit together; one such category (“crisis texting”) served as the foundation for this article. During weekly analyst meetings, themes were constructed from categories by reviewing data and grouping codes and quotations that aligned. Themes were presented and refined in consultation with the broader analytical team (including ART, LD, PCB, and JH). Given the small number of qualitative studies on experiences with CTSs, this study was designed to make broad interpretations regarding CTS experiences among young adults aged 18 to 25 years. Thus, although demographic information was collected, subgroup analyses (by race, sexual orientation, etc) were not conducted within this study. COREQ (Consolidated Criteria for Reporting Qualitative Research) guidelines were used to guide qualitative methodology reporting [35].

## Results

### Participant Demographics

Participants’ demographics are described in Table 1, and the method of communication for the interview is given in Multimedia Appendix 2.

The mean age was 22 (SD 2.28) years, with a range of 18 to 25 years. Regarding gender, participants most commonly identified as a boy or man (n=17, 44%), a girl or woman (n=14, 36%), or nonbinary (n=12, 31%). The majority (n=33, 85%) of participants identified as one or more minoritized sexual orientations (which we define here as bisexual, queer, lesbian or gay, asexual, pansexual, unsure, aromantic, or multiple sexual orientations). Participants were majority White (n=31, 79%) and non-Hispanic (n=32, 82%). Information regarding CTS use among participants can be found in Table 2.

**Table 1.** Participants demographics (n=39)

Characteristic	Participants, n (%)
Age (y)	
18‐19	10 (26)
20‐21	6 (15)
22‐23	12 (31)
24‐25	11 (28)
Gender^a^	
Boy or man	17 (44)
Girl or woman	14 (36)
Nonbinary	12 (31)
Gender fluid	4 (10)
Agender	3 (8)
None	2 (5)
Something else	1 (3)
Sexual orientation^a^	
Bisexual	11 (28)
Queer	9 (23)
Lesbian or gay	9 (23)
Asexual	6 (15)
Pansexual	6 (15)
Straight	6 (15)
Unsure	3 (8)
Aromantic	3 (8)
Race^a^	
White	31 (79)
Asian	8 (21)
American Indian or Alaska Native	3 (8)
Black or African American	2 (5)
Ethnicity	
Non-Hispanic	32 (82)
Hispanic	7 (18)
Geography	
Urban	18 (46)
Suburban	16 (41)
Rural	3 (8)
Something else	2 (5)

a Multiple choices were allowed; rows do not add up to 100%.

**Table 2.** Crisis text service use characteristics among participants (n=39).

Crisis text service use characteristic	Participants, n (%)
Crisis text service use	
Yes	26 (66)
No	13 (33)
Number of crisis text service encounters^a^	
1‐3	10 (28)
4‐6	7 (28)
7‐10	4 (16)
>10 times	4 (16)

a One participant chose not to disclose the number of times used.

Participants reported using 988, Crisis Text Line, the Trevor Project, and other local crisis lines. Participants’ use of crisis lines varied widely: some reported using them during periods of acute suicidal crisis, while others reported using them while experiencing more passive SI. Interview content focused primarily on CTS use for SI. When discussing their experiences with CTSs, most participants explicitly discussed using CTSs for SI, connecting their experiences or considerations to SI-related concerns. Participants sometimes spoke more generally about CTSs as a resource. Participant quotations mentioning the use of CTSs for other issues (eg, anxiety) were omitted from the analysis.

We generated 5 themes from our analysis. Our findings suggest that young adults with lifetime SI (1) see CTSs as having a unique role among crisis services, (2) value texting as a way to communicate, (3) dislike generic and impersonal interactions with CTSs, (4) view anonymity as serving dual purposes, and (5) experience varying effects from using CTSs for SI. Additional supporting quotes are in provided Table 3.

**Table 3.** Additional representative quotations illustrating study themes.

Theme	Supporting quotes
CTSs^a^ functioning uniquely among crisis services	“As for the other ones, they were especially helpful when I was younger (in high school). I was struggling a lot with my sexuality + going to a Catholic school in a socially conservative area, so I got a lot of affirmation from the text lines.” (Taylor) “If I had resources available, I would probably do something similar to last time as have friends and my partner kinda babysit me until I was doing better, otherwise I would definitely consider a crisis line and in the worst situation maybe talking to my therapist.” (Amelia)
Appreciation of texting as a communication mode	“I am more comfortable knowing that the person I’m reaching out to won’t be able to contact anyone I know or know where I live to send emergency services (I was more worried about this in the beginning but I still prefer it personally) because I felt like I could divulge the information I was comfortable sharing while still having privacy.” (Jazz) “It was easy, low-pressure, and pretty instantaneous. I never had to wait too long to be connected to someone. I hated phone calls, so there was no way I’d ever voluntarily call the 1-800 number; I also didn’t have to worry about someone overhearing me (this was especially important in high school). I could proofread whatever I wanted to say and send “stop” whenever I wanted without feeling guilty for hanging up.” (Taylor)
Dislike of generic and impersonal CTS interactions	“I’ve texted I think four times, it’s the same deal every time ‘this seems difficult, how have you been feeling?, distract yourself!’” (Wyn) “not necessarily dismissive because she did say ‘you’re brave for reaching out tonight’ and that jazz but I definitely wanted a bit MORE you know? just like some validation around the specifics of what I shared. kinda felt like she didn’t react to it or really fully read it in a way.” (Bernadette)
Anonymity as a motivator and vulnerability	“I remember when they rolled the 988, number out that a lot of people that were just kind of in my social circle, were like, Oh, don’t trust it. They’re just going to call the cops straightaway. And even though I’ve looked into it myself and realized that’s not the truth, I think just that fear has always just kind of been in the back of my head.” (Oliver) “It’s hard because you hear so many stories about mentioning suicide and then doctors just hauling you off (exaggerating) to a mental hospital, so I am hesitant about talking about it with them sometimes.” (Wyn)
“Mixed bag”: Variable perceived benefit of CTS within and across participants	“I’ve had some good and some bad experiences. I had a couple really nice experiences where I felt like I made a connection with the person and they were very supportive. But I also had a couple bad experiences where they took a long time to respond and when they did it was a very copy paste response and dismissive feeling.” (Jazz) “And, as a teen it made me feel slightly worse (perhaps obviously related to inability to communicate with full accuracy) as an adult they’ve helped me gain some clarity and provided feedback I needed to make certain changes in my life.” (Alex)

a CTS: crisis text service.

### CTSs Function Uniquely Among Crisis Services

Nearly all participants reported accessing multiple touchpoints within the crisis care ecosystem, including crisis lines, inpatient and outpatient services. Given the perceived accessibility and privacy of CTSs, young adults positioned them relative to other crisis services, often considering them a backup option or a beginner resource.

CTSs’ use as a backup or beginner resource was facilitated by participants’ ease of access to the service. Participants mentioned learning about, or being reminded of, crisis line numbers from student identification cards, television, and social media. Paul reported texting a crisis line because that’s what “the instructions on the suicidal ads told me to do.”

This ease, when compared to the lack of ease in accessing other services, enabled CTSs to be viewed as a backup resource. CTSs operated as a backup in 2 ways: to avoid burdening others and to be used during heightened SI. As Bernadette explained:

I think crisis text is kind of my backup option when I don’t wanna bother my other supports. I don’t wanna text my therapist in the middle of the night, sometimes I don’t feel like texting my mom or friends either when I’m really not feeling good mentally.

CTSs came to be seen as a “backup” for Bernadette because she preferred not to bother people in her life. This sentiment was a common motivator for CTS use among participants who feared what they viewed as the emotional burden of hearing SI disclosures. Others, like Wyn, were concerned about potential strain on relationships and explained, “since my suicidal ideation is here to stay, I don’t want my friends to get tired of me and my problems, so it’s a stranger or it’s nothing.”

In contrast to relational considerations, others framed CTSs as a backup specifically for moments of heightened SI. Maria, who had never texted a crisis line, shared that “If it were really an issue, I would talk to the hotline (although begrudgingly)” while Renee, who also had never texted, shared that she would only engage a CTS “if it came to that.”

Others reported perceiving CTSs as a beginner or gateway resource compared to other, more involved services. Taylor, who had used CTSs in college, “used the text lines to build up the confidence/understand the severity of needing to tell my therapist that I was suicidal,” demonstrating how CTSs functioned as practice for alternative services. Many participants described maturing service trajectories, in which they became more knowledgeable about which services worked for them over time, thereby altering service use. Others, like Willow, attributed her use of CTSs to being “fairly young and uninformed by other resources at the time.” Charlie, who had previously used CTSs, shared that he did not think he would be using CTSs in the future because he has “better resources to access now if I choose to.” Thus, young adults engagement of CTSs was shaped by their perception of the service as a highly accessible beginner and potentially backup resource.

### Appreciation of Texting as a Communication Mode

Participants’ appreciation for CTSs was motivated by comfort with texting, the ability to edit text messages, having time to think before sending a message, and feelings of privacy and anonymity.

Many participants reported using CTSs because they prefer text communication, especially for emotionally difficult disclosures such as SI, with some aiming to avoid spoken disclosures of SI. Lily described texting as “more comfortable and available,” and Oliver, who had never used a crisis line of any kind, reflected that he believed the difficulty of verbal disclosures of SI would lead him to choose a text service if he were to use one. He shared:

It’s hard to vocalize things when you’re…struggling with suicide….Like out loud to say, like, I’m struggling with this, where it’s a lot easier just to type it. So I would definitely use text.

Beyond general comfort with texting, participants also valued the ability to review and modify text messages when using CTSs, which was highlighted as a common reason for their use. Time to think and edit was especially valued in the context of SI disclosures, given the potential consequences of misunderstandings. Alex explained:

Texting…provided a level of disconnection. Kinda like a buffer between the general rhythm of a verbal conversation where immediacy is more or less expected, whereas pausing can be more easily interpreted as an indication that somethings wrong.

Jazz, speaking more broadly about texting crisis lines when he was in distress, explained a similar phenomenon, noting that phone calls stressed him out because of the requirement to “respond on the spot without really having time to think,” and that texting allowed him to type what he wanted and adjust it.

Another benefit perceived by participants was that texting further provided a sense of privacy from the people in their immediate surroundings, expanding the places where crisis services could be accessed and, consequently, where participants could obtain support for their SI. Ophelia had used CTSs on the bus, explaining that “no one really knows who you’re texting,” while Phoebe mentioned using CTSs “in the middle of a lecture.”

For some, privacy, conferred mostly through the silence of texting, was a matter of safety. Alex shared using CTSs “in abusive living situations where it would’ve been potentially dangerous for me to have a verbal conversation.” Others, like Charlie, while not explicitly mentioning safety concerns, mentioned using CTSs when they were younger and worried about being overheard disclosing SI. Paul shared that he chose CTSs over the phone because he knew his parents “would hear me if I called” and did not want them to know about his SI.

Thus, perceptions of CTSs and experiences with CTSs were heavily influenced by their technological features, which enabled convenience and privacy, shaping when, where, and how young adults were willing to seek support.

### Dislike of Generic and Impersonal Interactions

Although participants appreciated the technological features of CTSs, they expressed distaste for what they perceived as the generic, impersonal interactions they had with crisis counselors, characterized by scripting, a lack of attunement to their individual situations, and an overfocus on resource provisioning.

Discussing texting CTSs for SI, Wyn described CTS conversations as feeling “repetitive and scripted,” while Jazz described them as “the same deal every time.” Phoebe reported her experiences with CTSs, like “websites where their customer service thing is an automated chatbot.” Part of this, for Phoebe, was related to the scriptedness:

I’ve…talked to enough crisis counselors that I kind of know the general script or whatever. Especially with text, it really felt like there was a script…you could tell they were going through the motions of we need to ask this, and we need to say this.

Another way in which a sense of impersonality was created was when counselors failed to respond to texters’ unique situations or missed the point of a texter’s communication. Bernadette wanted “some validation around the specifics of what I shared” and explained that it “kinda felt like she didn’t react to it or really fully read it in a way.”

Beyond scriptedness, impersonality also emerged when texters were offered resources for SI too quickly or when they were not wanted. CTS conversations often conclude with the counselor helping the texter develop a safety plan, which may include offering resources. This flow may be misaligned with what some texters want, with some reporting a desire to feel understood or to have a place to “vent.” Therese questioned why “it just immediately goes to like, resources,” while Jazz shared an incident where the counselor had sent a resource “even though I told them that resource wouldn’t help my situation.” Similarly, Ray shared:

A lot of times like they’d send like breathing exercises, but I had reached out multiple times and I always got the same exact one….One time I brought up….Hey, is it okay that we don’t do breathing exercises because those tend to not really be super effective for me. And then they sent me breathing exercises!

Although participants valued the texting medium, they often felt the interactions were not sufficiently personalized or emotionally responsive, which affected both the quality of the experience and perceptions of CTSs’ effectiveness (discussed in the “*Hit or Miss”: Variable Perceived Benefit of CTSs Within and Across Participants* section).

### Anonymity as a Motivator and Vulnerability

Anonymity played a key role in participants’ decision-making and engagement regarding CTSs. For participants, anonymity served as a motivator for CTS use and as a potential vulnerability should it be compromised. The fear of losing anonymity because of an SI disclosure to CTSs, which could lead to parents discovering SI, forced psychiatric hospitalization, or law enforcement involvement, was always present, fostering hesitation to use CTSs.

Participants valued the ability to engage with CTSs privately. CTS users control how much information they share during conversations, and it is unlikely anyone will find out they used the service. Charlie described anonymity as “preferable” because he “would’ve been afraid to be caught as being suicidal” by his mom or school. Jazz, who had texted CTSs for SI, felt “more comfortable knowing that person I’m reaching out to won’t be able to contact anyone I know or know where I live.”

Others did not see CTSs as anonymous and worried that SI disclosures could lead to negative consequences, such as being recorded in a system that might penalize them later or escalate to unwanted services. Renee, who had not texted CTSs, shared such hesitation, illustrating how concerns about traceability potentially deter her from initiating contact, saying, “I had this fear…this [text conversation] is gonna go on, like some sort of record.” Jamie, who also had never texted a crisis line, worried, “about judgment or discrimination from a possible future employer if they somehow found out I had SI in the past.”

Often, these fears originated from stories on social media. Kim, who had texted a CTS, was “kind of nervous they were going to contact someone, and I would have to go to the hospital” because she has “seen people online text or call various hotlines and get sent to the hospital.”

Thus, anonymity encouraged CTS use by making the service feel private and discouraged some participants who feared it could be compromised if they disclosed SI.

### “Hit or Miss”: Variable Perceived Benefit of CTSs Within and Across Participants

Experiences and perceptions of CTS usefulness and impact varied widely among participants. Some participants who had not used CTSs expressed hesitation about their usefulness and viewed phone calls as more helpful. In contrast, participants who used CTSs repeatedly reported variable experiences, particularly regarding crisis counselor quality and CTSs’ impact on suicidal distress.

Participants who had not used CTSs often perceived them as less beneficial than similar resources. Micah, who had used crisis phone lines but never text lines, considered calling “more personal,” while Maria, who had used neither, similarly imagined talking on the phone as more helpful, given a “more sympathetic” counselor. Others echoed this preference for voice contact. Ray found a human voice comforting, sharing that it makes him feel like he is “being listened to a lot more than a text message.”

Among those who had used CTSs multiple times, participants described the service as “hit or miss” or “a mixed bag.” As a result, participants characterized the perceived effect of CTSs on their SI as neither strongly negative nor positive. Bernadette, who had texted CTSs about SI, noted what she saw as low-quality conversation from the counselor, said she “wanted a bit MORE” from the counselor. Others mentioned gaining some benefits from texting a CTS about SI but experiencing somewhat unexpected results. Taylor, who reported contacting CTSs about her SI, shared that she did not think CTSs made her significantly less suicidal; she found the conversation calming and helpful to “clear [her] head and vent.” Similarly, Wyn, when discussing the use of CTSs, said that “it’s been fine. I don’t feel like I get any help from the text lines but the process of recognizing I need help and asking for it usually centers me a little.” Nonetheless, no participant reported worsening distress as a result of CTS use.

Despite mixed or frustrating experiences, some participants still returned to CTSs during moments of distress. Wyn shared:

It sounds silly to go back and text again even though I’m saying they’re not very helpful but when I’m texting that number I’m not thinking “hm last time they weren’t very helpful” I’m just getting whatever help comes to mind first.

Wyn’s quotation highlights that repeated service use may be separate from perceived helpfulness or satisfaction. Overall, patterns of use and perceived impact varied both across participants and within the same individuals over time.

## Discussion

### Principal Findings

In this study, we report on the perceptions and experiences of using CTSs among young adults with SI. We constructed 5 themes to characterize this relationship, including how CTSs play a unique role among crisis services, appreciation of texting as a mode of communication, dislike of generic and impersonal interactions, the dual role of anonymity, and variable perceived benefit within and across participants. The findings suggest that CTSs play a critical role in young adults’ crisis care ecosystems and contain numerous areas for potential improvement to better serve suicidal young adults. Recommendations following these findings are highlighted in Table 4.

The findings suggest both positive and negative experiences with CTSs among young adults. Emerging evidence, mostly from pre- or postsurveys, has supported the ability of crisis lines to reduce short-term distress [20-24]. These findings add to this body of research by highlighting that both negative and positive experiences with CTSs are common and by illuminating the characteristics that shape them. Negative experiences with CTSs services, as reported by participants in this study, were typically linked to the quality of conversations with the crisis text counselor, which could feel robotic or insufficiently tailored to the individual. This finding aligns with others [24,25,36] reporting user dislike of robotic, depersonalized text counseling and a preference for personalized language in crisis counseling encounters. The findings further support research that describes relationships with providers as at the core of successful mental health crisis encounters [22,37-39].

Our findings of negative, neutral, or inconsistent CTS experiences suggest a need for improved conversational quality and greater consistency in CTS conversations. One possible pathway toward improved conversational quality is via enhanced crisis workforce training. Multiple participants reported feeling “rushed” into the later stages of the conversation including the provision of resources. In contrast to our findings, computational studies using data from text-based crisis counseling sessions have shown that conversations where less time is spent in rapport building and problem-exploration and more time is spent in later conversational stages (ie, goal identification, discovery of the next steps) are associated with *higher* satisfaction among CTS users [31,40]. Researchers have hypothesized that quicker movement into problem-solving and greater effectiveness are facilitated by *efficient*, *high-quality* rapport building. This rapport is marked by avoiding templated responses and being highly attuned to the conversational trajectory [39]. Thus, participants who reported feeling rushed into later stages may have been conversing with counselors who used poor rapport-building techniques. The conversational quality of CTSs could be improved by applying evidence-based crisis counseling strategies (eg, efficient rapport building and more time in later conversational stages) to conversations and incorporating them into counselor training.

**Table 4.** Recommendations for crisis text services informed by participant experiences.

Area	Recommendation
Crisis text service encounters	Apply evidence-based crisis counseling strategies (efficient rapport building, more time in later conversational stages) Enhance counselors’ expressions of understanding and empathy Minimize crisis counselors’ use of generic resources Prioritize rapid responses to users’ texts Emphasize tailored communication
Systems or policy	Strengthen US crisis text counselor workforce via competitive wages, remote work opportunities, mentorship, and supportive monitoring Bolster federal and state funding for crisis phone and text lines Enhance crisis counselor workforce training
Research	Examine the impact of increased disclosure regarding scenarios requiring escalation to emergency medical services or law enforcement Explore successful linguistic and conversational strategies for use within crisis counseling conversations

While many relational factors have been identified as constructive within therapeutic encounters, such as catharsis, positive relationship, therapeutic alliance, warmth, and trust [41,42], the evidence base for best practices for suicide prevention within text-based crisis counseling encounters is far more limited. More research is needed to understand the effectiveness and deployment of conversational and linguistic strategies in crisis counseling–specific CTS conversations. Areas suggested within this study by CTS users suggest that conversations could be improved by more tailored communication, including a reduction in the use of generic resources, reliably expedient responses, and deeper expressions of understanding and empathy.

Improving the quality of crisis counseling is a difficult task. Demand for crisis counselors increased following the 2022 launch of the 988 crisis line, and many US states have faced challenges staffing crisis counseling agencies at sufficient levels [43-45]. Moreover, reports of turnover and burnout among the crisis counseling workforce are high, and workers report varied levels of training [46]. Thus, improving conversational quality may require enhanced financial support for the crisis line workforce through mechanisms such as competitive wages, remote work, mentorship, supportive monitoring, and ongoing training [43]. Continuing financial support for US crisis line operations and ongoing evaluation will remain crucial.

Although the impact of CTSs on SI as reported by participants varied, many participants in this study were highly appreciative of CTSs. As a service, CTSs were nearly uniformly reported to be easily accessible for disclosure of SI and for receiving crisis counseling. Consistent with other studies [20-22,47,48], many participants reported short-term improvements in mental distress and appreciation of the temporal and spatial flexibility afforded by CTSs. These findings enhance our understanding of the technological features of CTSs that motivate young adults to choose them over other options, such as their silent nature, a sense of privacy and anonymity, and the ability to think and edit before sending a message. On the other hand, young adults also hinted at an alternative consequence of the technological features of CTSs, whereby the silent nature and ability to edit responses enabled them to calibrate their levels of suicidal disclosure, allowing them to receive the desired level of care while avoiding EMS or law enforcement involvement. This phenomenon is described in detail elsewhere [49].

Young adults’ regard for the technological features of CTSs, combined with the fear of negative consequences from disclosure, supports the notion that young adults may potentially be drawn to CTSs due to an enhanced sense of control [24,28,30]. Since young adults may be attracted to technological features that enable a sense of agency, mental health crisis services could consider how these characteristics, or more abstractly, the ability to exert more control, can be meaningfully incorporated across the crisis ecosystem. This could incentivize greater engagement with these services and increase the likelihood that young adults reach out for SI support.

The findings provide further insights into how young adults compare calling with texting a crisis line, revealing that while texting is considered more convenient and accessible, it is not universally preferred over calling. Although scholars have reported that young adults prefer texting over calling [20], our findings suggest these preferences are not homogeneous; some young adults reported a preference for phone calls, considering them more personal and effective. This work further underscores the necessity of robust federal and state financial support to sustain multiple modes of high-quality crisis lines, especially in light of recent decreased federal funding for specialized LGBTQ+ crisis lines [50].

While this study did not focus on barriers to CTS use, the findings highlight factors that influence these decisions. Barriers can be classified into 2 categories: obstacles to first-time use and obstacles to repeated use. For those who have not used CTSs, barriers included beliefs in stronger effectiveness of other resources and negative experiences shared on social media or by friends. For previous users, future use was deterred by conversations perceived as ineffective or low-quality. While social media narratives regarding perceptions of involuntary hospitalization may not accurately reflect crisis services protocols, these narratives are nonetheless influential in shaping young adults’ SI-related help-seeking behavior.

Addressing the barriers to use in these 2 different cases may require different strategies. First, barriers to first-time use could be mitigated through campaigns that provide potential CTS users with information about escalation policies and who crisis lines serve. Additionally, the finding that crisis text lines may be used as beginner resources further suggests that many users will be unfamiliar with CTSs and policies, making information about the service even more essential. Despite many (~42%) US adults having heard of 988 [51], willingness to use crisis lines remains low, with only 1 in 4 (25.4%) young adults reporting willingness to use one [52]. Thus, what may be lacking is not awareness but rather information regarding the purpose of CTSs, intended audience, and protocols, especially in relation to EMS or law enforcement involvement. Despite fewer than 1% of crisis line calls resulting in emergency services deployment [53], the fear of medical or law enforcement involvement remains a significant barrier and a persistent cultural narrative in young adults’ decision-making about health service use.

Secondly, given that SI often recurs over time, and that 37% to 40% of texters are repeat users [54,55], it is crucial to consider barriers that prevent individuals from using the service after initial use. One possible pathway to counterbalance this barrier is through improved conversational quality which would encourage rather than discourage repeated use.

### Strengths and Limitations

This study provides a qualitative account of how young adults with SI perceive and engage with CTSs. This study is strengthened by its inclusion of a text message interviewing option, which may have expanded its reach to those who are uncomfortable with traditional face-to-face interviewing. The study findings should be interpreted considering key limitations. To sample participants, we used convenience sampling; thus, these findings should not be considered generalizable. Despite efforts to racially diversify the study sample, it remains majority White. While this is representative of Oregon, further research is needed to understand how minoritized racial groups at elevated risk of suicide, including American Indian, Alaska Native, Native Hawaiian, and Black young adults [1], perceive and use CTSs. Despite these limitations to study generalizability, we believe concepts discussed, such as dislike of nonspecificity and appreciation of the technological features of CTSs, may be conceptually resonant with similar populations, services, or technologies.

Although this study did not explicitly recruit LGBTQ+ young adults, 85% (n=33) of participants were LGBTQ+. This may be due to Oregon having the highest percentage of LGBTQ+ people among US states [53], 23% of Generation Z young adults (all participants were born between 1997 and 2012, thereby designating them Generation Z) identifying as LGBTQ+ [56], and LGBTQ+ young adults experiencing SI at twice the rates of non-LGBTQ+ peers [2]. Given the higher-than-expected percentage of LGBTQ+ participants in this study, the transferability of these findings to other groups of young adults with lower rates of LGBTQ+ identification is limited. It is possible that some aspects of these findings, in particular, the appreciation of the silent texting medium and anonymity, may be more influential for young adults who are managing a marginalized sexual orientation or gender alongside their SI. Future work is needed to understand how sentiments about CTSs may differ across population subgroups.

Finally, interviews were conducted in three different modes (phone, Zoom, and text), which likely impacted data quality. Within this study, we found that data across modes, while linguistically different (Zoom and phone interviews tended to include more words and detail), were thematically similar. Similar levels of forthrightness were observed across modes. Our experiences conducting multimodal data collection in this study are discussed in detail elsewhere [57].

### Conclusions

The study findings indicate that CTSs play a crucial yet imperfect role in young adults’ crisis service ecosystems. CTSs are perceived as accessible and often helpful for SI. However, the quality of conversations was reported as inconsistent, and some young adults expressed fear of negative consequences from disclosing SI. These findings support the potential benefit of policy and programming that increase the quality and consistency of CTS conversations. CTSs may be better able to serve suicidal young adults through additional support for ongoing assessment, evaluation, and quality control, possibly including the development and implementation of evidence-based guidelines for crisis counselors. These improvements could potentially enable CTSs, which are widely used resources, to better meet the needs of young adults experiencing suicidal thoughts, reducing the likelihood of suicide.

## Supplementary material

10.2196/89836 Multimedia Appendix 1 Interview guide.

10.2196/89836 Multimedia Appendix 2 Interview mode and participants’ pseudonyms.

## Abbreviations

COREQ: Consolidated Criteria for Reporting Qualitative Research
CTS: crisis text service
EMS: emergency medical services
LGBTQ+: lesbian, gay, bisexual, transgender/transsexual, queer, and other minority sexual orientations and gender identities
SI: suicidal ideation
