# Supplementary material for: Experiences and Perceptions of Crisis Text Services: Interview Study Among Young Adults With Suicidal Ideation
Source: JMIR Ment Health. 2026 Aug 26;13:e89836. doi: 10.2196/89836 (PMC13515342; doi:10.2196/89836)
Supplement: Supplementary file 1 [file mental-v13-e89836-s001.docx]

## Appendix Item 1: Final Interview Guide

*ALL PARTICIPANTS*

- Ok, so to start, could you describe a typical day for you right now?

**Suicidal Ideation**

- For this study, I’m particularly interested in how people seek help with suicidal ideation or thoughts. Mind sharing your history of suicidal thoughts?
- How do you decide when and how to get support?

**Experiences with help-seeking overall**

- Have you encountered any barriers or challenges when accessing support for SI? Internal or external?
- Can you share your history with therapists?
- Do you talk to them about SI?
- How do you decide when to contact them if you’re having SI vs doing other things?
- Have you talked to your mental health provider about your use of crisis lines?
- Have you ever gone to the ER for suicidal thoughts?

**Privacy and control**

- How important is privacy to you when considering seeking help for suicidal thoughts?
- Can you describe any concerns or hesitations about disclosing SI to crisis service providers in terms of maintaining your privacy?
- What do you think about privacy when using tech-based resources like crisis text lines?
- Some people have told me they worry about losing autonomy or control in crisis settings, and others think that that’s just what should happen. Where do you come down on that?
- Can you share any experiences where you felt your autonomy was respected or disregarded by crisis service providers?
- OPT: How do you navigate the balance between seeking support from crisis services and maintaining a sense of autonomy and independence in managing your mental health?

**Emerging adulthood**

- How has your decision-making about where you seek help for SI changed over time?
- Has it gotten easier or harder to manage your SI over time?
- How has your view of crisis services changed as you’ve become more familiar with services and yourself over time?
- Can you describe any recent life changes or transitions that have made you reconsider how you seek help for SI?
- Have you had any changes in your relationships that have impacted how you seek help for SI?
  - With parents?
  - With friends?
- How does being more independent (or x) change how you seek help?
- How do you balance the need for independence (or x) and self-reliance (or y) with recognizing when to seek help for SI?
- How does your financial situation relate to what sources you use for help with SI?
- Around this time, it’s common for people to become more financially independent from their parents. How have you and your parents decided who pays for what regarding mental healthcare?
- Could you describe your dialogue with your parents about money and mental healthcare?

**Intersectionality**

- You said you identify as queer; how does that impact how you seek help for SI?
  - How has that changed over time?
- How do you think your race impacts how you seek help for SI, if at all?
- Can you share any cultural beliefs or practices related to help-seeking for suicidal ideation that influence your approach to seeking support?
- How do you think your cultural background or identity influences your attitudes toward seeking help for suicidal ideation?

*TEXTERS ONLY*

**Experiences with CTS FOR SI**

- What makes you text a crisis line instead of using any other resource?
- Thinking back to the last time you used a crisis text line, what caused you to reach out?
- Can you think of a time you used a crisis line that stands out?
- What happened?
- How did it go?
- I’m curious about your physical surroundings. Where were you?
- What did you hope to get out of the conversation?
- How did the conversation end?
- Have you ever had a bad experience texting a crisis line? What happened?
- Is there anything about crisis text lines that makes you hesitant to use them?

**Perceptions of text-based technology**

- What is it like talking about suicide and mental health by text?
- What do you like about texting about suicide and mental health?
- Is there anything you don’t like about texting about suicide and mental health?
- What features or functionalities of crisis text do you find most helpful for discussing suicidal thoughts?
  - Least helpful?
- In terms of expressing how you’re feeling, do you think crisis text is easier or harder compared to other resources?
- Why did you text a crisis line instead of calling one?
- Do you have an opinion on crisis text counselors talking to more than one person at a time?
- When you text a crisis line, are you doing anything else on your phone?
- How similar or different is texting a crisis line compared to texting a friend or someone you know? /anonymous?
- And sort of related to that, texting a crisis line means different things to people. Does it feel like a big deal to text a crisis line, or is it no big thing?
- And earlier on you mentioned that texting is not the best way to talk about SI. And what do you think is the best way to talk about SI?

**Counselor**

- Do you think much about the counselor you’re talking to on the other line?
- Did you wonder about the counselor's emotions or thoughts during your conversation?
- How did you imagine the counselor on the other end of the line? Can you describe them?

**Impact of crisis text**

- You mentioned that you’ve used crisis text when you’re having SI. What impact have those conversations had on your SI?
- Where do you see crisis text fitting into your mental healthcare overall?
- Now that you’re living on your own, would you still use crisis text or something else?
  - How has using crisis text changed the way you think about your SI?XTERS:
- Since you’ve never texted a crisis line but heard of them, what’s stopped you?
- What have you heard about them?
- If you didn’t think it would help, what makes you say that?

*ALL PARTICIPANTS*

**Closing**

- Looking back at your experience with SI and getting help, are there any events that we haven’t talked about that really stand out to you?
- Those are all the specific questions I have. Is there anything else you’d like to add to help me understand your experiences with using when you’ve had SI?
- Ok, so that’s all the topical questions I have. I like to end interviews with one positive question. What do you view as a strength of yours?
