# Supplementary material for: Experiences and Perceptions of Crisis Text Services: Interview Study Among Young Adults With Suicidal Ideation
Source: JMIR Ment Health. 2026 Aug 26;13:e89836. doi: 10.2196/89836 (PMC13515342; doi:10.2196/89836)
Supplement: Supplementary file 2 [file mental-v13-e89836-s002.docx]

**Multimedia Appendix 2: Interview Mode and Participants Pseudonyms**

| Interview Mode (n=39) | Participant Pseudonyms |
| --- | --- |
| Text (n=19) | Alex, Amber, Amelia, Bernadette, Cameron, Charlie, Dmiitri, Finn, Jamie, Jazz, Kim, Leo, Lily, Maria, Morgan, Sloane, Taylor, Willow, Wyn |
| Phone (n=12) | Avery, Brianne, Jade, Micah, Nate, Paul, Phoebe, Renee, Silas, Tabitha, Thomas, Tiff |
| Zoom (n=8) | Byron, Jasper, Noah, Oliver, Ophelia, Ray, River, Therese |
